# Photodynamic Therapy Using a New Painless Light-Emitting Fabrics Device in the Treatment of Extramammary Paget Disease of the Vulva (the PAGETEX Study): Protocol for an Interventional Efficacy and Safety Trial

**DOI:** 10.2196/15026

**Published:** 2019-12-03

**Authors:** Fabienne Lecomte, Elise Thecua, Laurine Ziane, Pascal Deleporte, Alain Duhamel, Cyril Maire, Delphine Staumont-Salle, Serge Mordon, Laurent Mortier

**Affiliations:** 1 U1189 - Image Assisted Laser Therapies for Oncology, Inserm Centre Hospitalier et Universitaire de Lille Université de Lille Lille France; 2 EA 2694 - Santé Publique: Epidémiologie et Qualité des Soins, Unité de Biostatistiques Centre Hospitalier et Universitaire de Lille Université de Lille Lille France; 3 Department of Dermatology Centre Hospitalier et Universitaire de Lille Lille France

**Keywords:** photodynamic therapy, extramammary Paget disease of the vulva, light emitting fabrics, methyl aminolevulinate

## Abstract

**Background:**

Extramammary Paget disease of the vulva (EMPV) is a rare skin disorder commonly seen in postmenopausal Caucasian females that appears clinically as red, eczematous, pruriginous, and sometimes painful lesions. Although most cases are noninvasive, EMPV may be associated with an underlying or distant adenocarcinoma. EMPV has a chronic and relapsing course. The reference treatment is based on local surgical excision with negative margins. However, disease frequently extends far from the visible lesion, and surgical margins are frequently positive. Topical photodynamic therapy (PDT) is an established treatment modality for various dermatooncologic conditions. For example, red light irradiation with the Aktilite CL 128 and Metvixia (Galderma SA) as a photosensitizing molecule is a conventional protocol approved and widely used in Europe for PDT treatment of actinic keratosis, but this treatment is not yet widely used for EMPV because it has never clearly been demonstrated and is very painful.

**Objective:**

The aim of the study is to investigate the efficacy and safety relating to the medical device PAGETEX as a new painless PDT device using Metvixia in the treatment of vulvar Paget disease. The primary end point is the disease control rate at 3 months in 30% of the patients included, defined as stability, partial response, or total response, considering the extent of the lesion. Secondary end points are the disease control rate at 6 months, patient quality of life, level of pain experienced by the patient at each PDT session, severity of erythema, presence of protoporphyrin IX in Paget cells after each PDT session, and overall satisfaction level of the patient.

**Methods:**

The trial is an interventional, exploratory, simple group, nonrandomized, and single center (Lille University Hospital) study. Twenty-four patients will be included according to Simon’s optimal plan. Therapeutic procedure is based on a cycle of two PDT sessions with the PAGETEX medical device at 15-day intervals (Metvixia incubation during 30 minutes and 635 nm red light illumination with a low irradiance for 2 hours and 30 minutes for a total fluence of 12 J/cm²). At the assessment session, 3 months after inclusion, if the control of the disease is partial or null, the patient will complete another cycle of two PDT sessions. A final evaluation will be performed in all patients at 6 months. Analyses will be performed using SAS version 9.4 software (SAS Institute Inc). The characteristics of the patients at baseline will be described; qualitative variables will be described by numbers and percentages, and quantitative variables will be described either by the mean and standard deviation for Gaussian distribution or by the median and interquartile range (ie, 25th and 75th percentiles). The normality of the distributions will be tested by a Shapiro-Wilk test and checked graphically by histograms.

**Results:**

First patient was included in September 2019 and clinical investigations are planned until August 2022. The final results of this study are expected to be available in January 2023.

**Conclusions:**

This clinical trial aims to evaluate the efficacy and safety of a new PDT protocol for the treatment of EMPV. The PAGETEX device could become the treatment of choice if it is effective, painless, and easy to implement and use in hospitals.

**Trial Registration:**

ClinicalTrials.gov NCT03713203; https://clinicaltrials.gov/ct2/show/NCT03713203

**International Registered Report Identifier (IRRID):**

PRR1-10.2196/15026

## Introduction

Extramammary Paget disease of the vulva (EMPV) is invasive in 20% of cases. If the invasion is less than 1 mm, the evolution is the same as that of noninvasive lesions. Beyond 1 mm, or when there is an underlying carcinoma, the prognosis is worse with a risk of remote lymph node metastases [1].

The disease is usually observed in Caucasian women over age 70 years and is revealed by pruritus. The lesion is red and ulcerated with an irregular surface and is often mistaken for an eczematized inflammatory lesion. The disease sits on the lips and sometimes bilaterally extends to the perianal region. The actual extension may be more important than the clinical lesions, and multiple biopsies can be used to evaluate the extent of the lesions to guide the surgical procedure.

Histologically, the disease is characterized by intraepidermal proliferation of large epithelioid cells with abundant and clear granular or vacuolized cytoplasm. The reference treatment is based on surgical excision. This surgical treatment is facilitated by new techniques in plastic surgery and anesthesia that will limit postoperative sequelae. Unfortunately, besides the fact that the surgery is sometimes mutilating, local recurrences are also very frequent (up to 45% of the cases) even if the margins of excision are healthy [2]. Since the prognosis of EMPV is good in the absence of invasive zone or underlying adenocarcinoma, the treatment of choice is surgical removal of the plaque with margins of 1 cm. Alternative conservative treatments such as laser therapy, radiotherapy, chemotherapy, and the application of topical treatments (imiquimod, corticosteroids) or topical photodynamic therapy (PDT) offer an interesting alternative, but they are mostly invasive and painful [3].

It is therefore essential to have a painless therapeutic alternative allowing a complete remission rate without functional or aesthetic sequelae. PDT is a technique based on the combination of photosensitizing molecules (PS) capable of focusing in tumor cells and a focused light of an appropriate wavelength (PS dependent). The combination of these two factors specifically targets the injured tissues and destroys them. PS is administered topically and is more or less selectively concentrated in the damaged tissue by irradiation that is used at a wavelength appropriate to the photosensitizer and which leads to necrosis or apoptosis of the cells. The photosensitizer used most often is a precursor of porphyrin, 5-aminolevulinic acid (5-ALA), and recently its methylated form, methyl aminolevulinate (MAL), was authorized in France under the name Metvixia. Conventional PDT usually includes incubation of 5-ALA or MAL for several hours. Applied to the skin, these prodrugs are converted endogenously by the biosynthetic pathway of heme in protoporphyrin IX (PpIX) and other intermediate photosensitive porphyrins, leading to a high and selective accumulation of PplX in the target lesion. Production of PpIX is visible by fluorescence when lesions are illuminated with a blue light. Abnormal cells accumulate more photosensitizer than normal cells. Illumination of these cells, using an appropriate light source, leads to apoptosis and selective necrosis of tumor cells, while sparing healthy adjacent tissues [4,5].

According to the product characteristics summary, Metvixia has been approved for use in combination with red light, so MAL-PDT has recently been introduced in clinical trials as a therapeutic option in EMPV [6-9]. The mode of delivery of light is one of the major concerns in PDT. Light sources marketed and used for the photodynamic treatment of skin lesions are generally flat, while the surfaces to be treated are mainly curvy. Thus, Moseley [10] showed that two commercialized LED devices did not provide a homogeneous light, and he demonstrated that the fluence rate could be 30% lower at a distance of only 2 cm than that delivered in the central zone. To overcome this disadvantage, the development of a flexible light source appears to be an interesting solution. The development of such technology, incorporating optical fibers into a flexible structure that emits fractional light, appears to provide a therapeutic solution for nonplanar anatomical surfaces such as curved surfaces and body extremities. A PDT device incorporating light-emitting fabrics (LEFs) is in use in several clinical studies [11-13] run by the Lille University Hospital for the treatment of actinic keratoses, and the first results seem to be promising, particularly with regard to the pain felt by patients.

The medical device PAGETEX consists of a textile diffuser support incorporating LEFs to diffuse light from a laser source. The complete device constitutes a source of nonlaser optical radiation and makes it possible to deliver a diffused and homogeneous illumination on vulvar lesions of the EMPV with an objective to perform an effective and painless PDT treatment on the affected region. This clinical protocol aims to evaluate the efficacy and safety of a new PDT protocol for the treatment of the EMPV.

## Methods

### Trial Design

The study is interventional, exploratory, simple group, nonrandomized, and single-center. A total of 24 patients will be included according to Simon’s optimal plan: 8 patients will be included in a first step and 16 other patients in a second step if and only if the test is not stopped in step 1.

### Setting

The study will be conducted at the Lille University Hospital, Lille, France, in the department of dermatology over a period of 30 months until the end of August 2022. Twenty-four patients will be recruited within 24 months and followed for 6 months.

### Device

**Figure 1:**
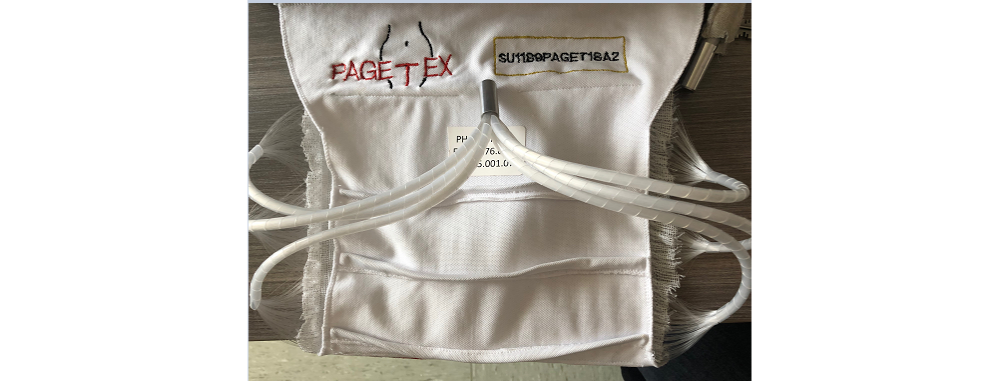
PAGETEX light diffuser support.

**Figure 2:**
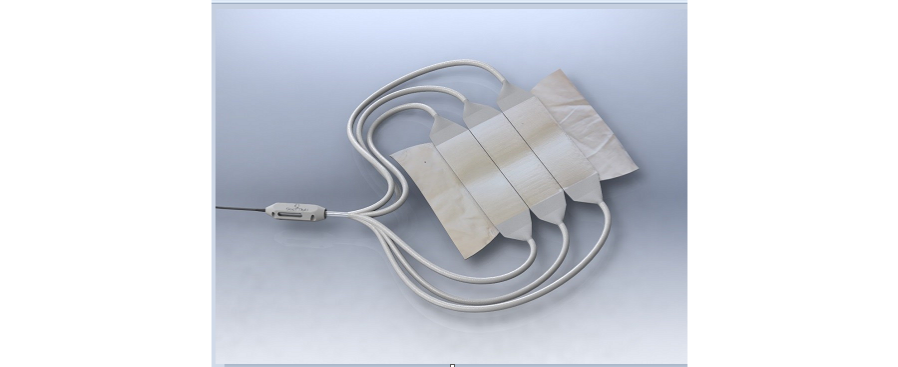
PAGETEX light emitting fabrics.

**Figure 3:**
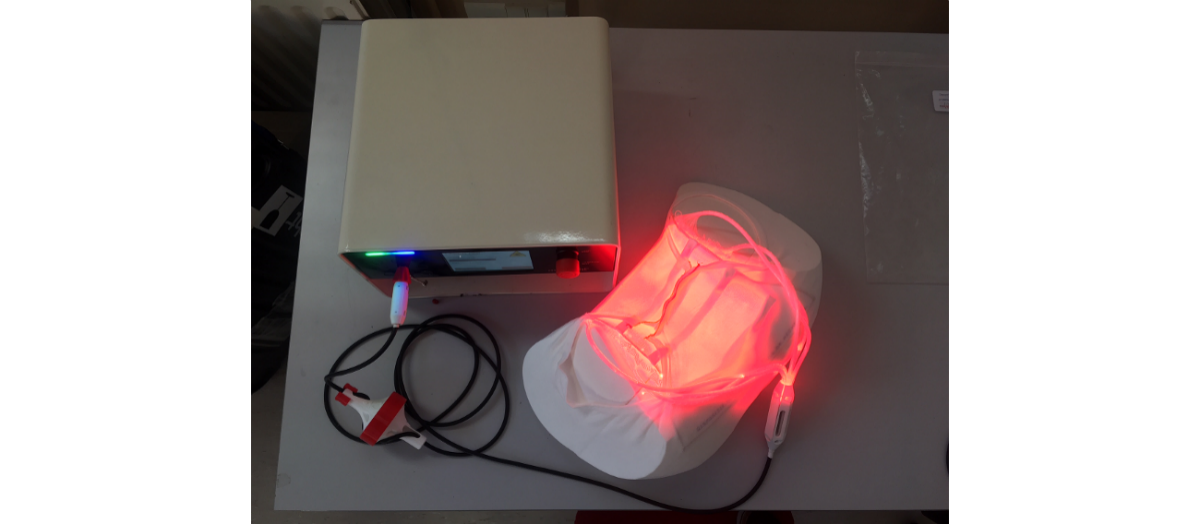
Complete device with medical panty, light diffuser support and laser source.

PAGETEX is a new illumination device consisting of a light diffuser support (Figure 1) incorporating three LEFs (Figure 2) to diffuse a red light from a medical laser source. Each LEF is connected to a 635 nm laser source (ML7710-630-EID707788 medical laser system, Modulight Inc), which is set to deliver a light dose of up to 12 J/cm². This light dose is controlled by an Ophir PD300 photodiode sensor connected to an Ophir Laser Star Bright power meter (Ophir Optronics Solutions Ltd). The light diffuser support is positioned on the vulva area and held with a medical panty (Figure 3).

### Participants

To be eligible for the study, patients must meet all of the inclusion criteria described in Textbox 1. Patients cannot have any of the exclusion criteria.

### Study Objectives and Outcomes

The main aims of the study are the efficacy of the device based on the disease control rate (stable, partial, or complete) considering the extent of the lesion at 3 months in 30% of the patients included and safety of the device based on the evaluation of adverse and serious adverse events.

Key secondary objectives are the disease control rate at 6 months, evolution of the quality of life, level of pain during PDT session, presence of Paget cells after staining of biopsies, presence of fluorescence in selected lesion after each PDT session, and evaluation of the tolerability and overall satisfaction level of the patient. Table 1 below details study objectives and outcomes.

**Selection criteria.**

Inclusion criteria:

- Women over age 18 years
- Diagnosis of noninvasive primary or recurrent after surgical resection Paget disease of the vulva
- Extramammary Paget disease of the vulva confirmed by biopsy within 1 year

Exclusion criteria:

- Invasive Paget disease
- Underlying adenocarcinoma
- Treatment with imiquimod 5% cream in the last 3 months
- Photodynamic therapy used to treat extramammary Paget disease of the vulva lesions in the last 3 months
- Use of photosensitive agents in the last 3 months
- Allergic or hypersensitivity to methyl aminolevulinate or any of the other ingredients in this medication (propyl p-hydroxybenzoate, cetostearyl alcohol, methyl p-hydroxybenzoate)
- Allergic or hypersensitivity to peanut or soya due to the presence of peanut oil in Metvixia
- Diagnosis of porphyria or immunity disorders (HIV, transplantation)
- Treatment with topical corticosteroids on the affected area in the last 3 months

**Table 1:** Outcomes and descriptions.

Outcomes		Inclusion^a^	PDT 1^b^	PDT 2^c^	Evaluation M3^d^	PDT 3^e^	PDT 4^f^	Evaluation M6^g^
**Primary outcomes**								
	Evaluation of lesion area and aspect by investigator and a blinded independent committee of physicians from standardized photographs taken during inclusion	x			x			x
	Disease control rate (stable, partial, complete response)				x			x
	Local tolerance (adverse event, serious adverse event, concomitant treatments)		x	x	x	x	x	x
**Secondary outcomes**								
	Evaluation of pain: visual analog scale graduated from 0 (no pain) to 10 (unbearable pain)		x	x		x	x	
	Severity of erythema (chromametry)	x			x			x
	Quality of life and satisfaction (DLQI^h^, FSFI^i^, SF-36^j^, and HADS^k^)	x			x			x
	Satisfaction questionnaire							x
	Presence of Paget cells: Positive/negative biopsy	x			x			x
	Presence of protoporphyrin IX in selected lesion (fluorescence detection)		x	x		x	x	

^a^Inclusion: D0.

^b^PDT 1: photodynamic therapy within 30 days or at D0.

^c^PDT 2: photodynamic therapy 15 days after PDT 1 (±2 days).

^d^Evaluation M3: D0 + 3 months (± 7 days).

^e^PDT 3 (optional): photodynamic therapy within 30 days after M3.

^f^PDT 4 (optional): photodynamic therapy 15 days after PDT 3 (±2 days).

^g^Evaluation M6: D0 + 6 months (±7 days).

^h^DLQI: Dermatology Life Quality Index.

^i^FSFI: Female Sexual Functioning Index.

^j^SF-36: 36-item Short Form Health Survey.

^k^HADS: Hospital Anxiety and Depression Scale.

### Sample Size

A minimum efficacy of 30% was set, and efficacy of 60% is expected to be achieved [14]. The hypotheses tested are P≤0.30 (H0) and P≥0.60 (H1), where P corresponds to the rate of patients with disease control at 3 months. Based on an optimal 2-step Simon plan (5% unilateral test), a total of 24 patients is required to test these hypotheses with a power of 80%: 8 patients are included in a first step, and the trial is stopped if the number of patients with a disease control at 3 months is less than or equal to 3; the PAGETEX device is considered not sufficiently effective. Otherwise, 16 additional patients are included in a second step. The PAGETEX device is not considered sufficiently effective if, on the 24 patients included (step 1 + step 2), the number of patients with a disease control at 3 months is less than or equal to 10.

### Allocation and Randomization

There is no randomization; all patients receive PDT treatment with the PAGETEX device.

### Implementation and Blinding

The study is not concerned with blinding as it is an uncontrolled clinical trial on a single group of patients receiving the same PDT treatment. However, to minimize the bias of investigator evaluation, an independent medical committee will evaluate the type of clinical response (stable, partial, complete, or no response) obtained at 3 and 6 months from standardized lesion photographs. Data will also be analyzed without blinding.

### Intervention

The course of the study is based on the protocol of PDT treatment with the Aktilite CL 128 (Galderma SA) applied in the dermatology department, to which have been added the investigation procedures specific to our research.

**Figure 4:**
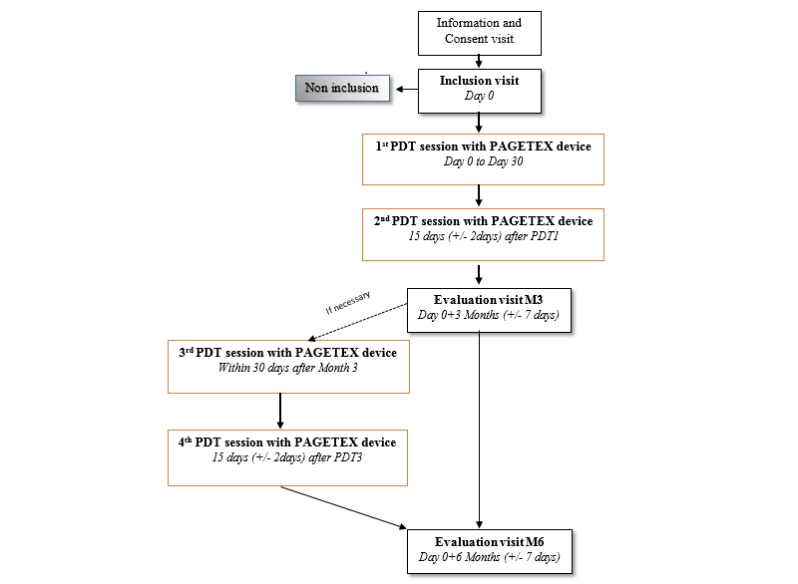
Progress of study visits. M: month; PDT: photodynamic therapy.

As shown in the flowchart (Figure 4), after inclusion visit, patients who meet all the criteria for inclusion and none of exclusion criteria are invited to come to the investigation site for 2 sessions of PDT and one evaluation visit at month 3. If necessary and if the disease control rate is not sufficient, patients are retreated with two sessions of PDT. A final visit at 6 months is made for all subjects in order to define the disease control rate.

### Preparation and Treatment of Lesions

At the inclusion visit, the investigator evaluates the appearance of the patient’s skin and the extent of the lesion. The largest vulvar lesion is selected, photographed, and measured with the Fotofinder dermoscope (Fotofinder Systems Inc) in natural light to define the basic extension of the disease before treatment. To confirm the presence of Paget cells and noninvasive EMPVA, a biopsy (3 mm in diameter and 2 to 3 mm deep) of the selected area is performed or results are obtained if the patient has had a biopsy within 1 year. The investigator grades the erythema, and pigmentation of the lesion is measured with the CR-400 Chroma Meter (Konica Minolta Sensing Europe BV). Colorimetric data and photograph of the lesion will be used as a basis for evaluation of disease control rate.

During PDT sessions, Metvixia is applied with a spatula on the selected vulvar lesion (approximately 1 mm thick and over an area of 5 to 10 mm of normal skin surrounding the lesion). The treated area is covered with a transparent occlusive dressing for 30 minutes. Then the light diffuser support is installed and retained by a medical textile panty. Illumination with 635 nm red light (12 J/cm²) is applied for 2 hours and 30 minutes. Pain is measured with a visual analog scale, and adverse events are collected.

At the evaluation visits, 3 and 6 months after initial treatment, the investigator and a blinded medical committee evaluate the disease control rate (stability, partial, or total response) by comparing the evolution of the selected lesion in terms of color and measures between the current visit and the first one. Table 2 shows all study procedures (exams and acts) performed during the trial.

**Table 2:** Study procedures and measures.

Action	Inclusion^a^	PDT 1^b^	PDT 2^c^	Evaluation M3^d^	PDT 3^e^	PDT 4^f^	Evaluation M6^g^
Informed consent	x						
Medical examination	x	x	x	x	x	x	x
Selection criteria	x						
Photographs of lesions under natural light	x	x	x	x	x	x	x
Biopsy	x			x			x
HCG^h^ pregnancy test		x	x		x	x	
Erythema gradation	x			x			x
Erythema measurement with CR-400 Chroma Meter	x			x			x
DLQI^i^, FSFI^j^, SF-36^k^, and HADS^l^ questionnaires	x			x			x
Application and incubation of Metvixia		x	x		x	x	
Illumination process		x	x		x	x	
Photographs of lesions under fluorescent light		x	x		x	x	
VAS^m^ measurement of pain		x	x		x	x	
Evaluation of tolerance/AE^n^/SAE^o^		x	x	x	x	x	x
Patient satisfaction							x

^a^Inclusion: D0.

^b^PDT 1: photodynamic therapy within 30 days or at D0.

^c^PDT 2: photodynamic therapy 15 days after PDT 1 (±2 days).

^d^Evaluation M3: D0 + 3 months (± 7 days).

^e^PDT 3 (optional): photodynamic therapy within 30 days after M3.

^f^PDT 4 (optional): photodynamic therapy 15 days after PDT 3 (±2 days).

^g^Evaluation M6: D0 + 6 months (±7 days).

^h^HCG: human chorionic gonadotropin test.

^i^DLQI: Dermatology Life Quality Index.

^j^FSFI: Female Sexual Functioning Index.

^k^SF-36: 36-item Short Form Health Survey.

^l^HADS: Hospital Anxiety and Depression Scale.

^m^VAS: visual analog scale.

^n^AE: adverse events.

^o^SAE: serious adverse events.

### Variables and Data Collection

Collected data consisted of demographic data, medical history reviews, previous medical and surgery treatments, and assessments of the subjects’ skin erythema. For female subjects of childbearing age, a urine pregnancy test is performed at screening and before each PDT treatment. Visual analog scale of pain and questionnaires (Dermatology Life Quality Index [DLQI], Female Sexual Functioning Index [FSFI], 36-item Short Form Health Survey [SF-36], Hospital Anxiety and Depression Scale [HADS] and satisfaction) are used.

### Data Management

The data are collected through a case report form and saved in an electronic file (database). All participants receive a trial identifier, and only the investigator knows the personal details. The sponsor’s monitor plans several monitoring visits, after the first inclusion in the study site location and periodically to assess data quality and study integrity. The sponsor’s monitor reviews study records and directly compares them with source documents, discusses the conduct of the study with the investigator, and verifies that the facilities remain acceptable. The monitoring of the trial is carried out according to the monitoring plan. A planning meeting with the principal investigator is held before the start of the trial. During the trial, several checkpoints are defined, including the presence of signed informed consent forms obtained by the investigator, respect of the inclusion and exclusion criteria, reporting of any adverse events, and the monitoring of all steps of patient follow-up. At the end of the trial and once the final analysis is completed and validated, all the files are sealed and archived according to specific procedures in a secure location in the sponsor clinical research department.

The trial support unit coordinates data management. The database is stored and secured on the network of Lille University Hospital. Before the closeout of the database, data monitoring is performed using SAS software (SAS Institute Inc) based on consistency rules set with the project manager (eg, missing data, outliers, and inconsistency between several variables). The data are analyzed in the Unit of Methodology, Biostatistics and Data Management of Lille University Hospital (UMBD). Only the investigator participating in the study or a collaborator designated by the physician and participating in the study may modify the data. The data concerning this study are archived for a minimum period of 15 years from the end of the research or its early termination without prejudice to the laws and regulations in force.

### Statistical Methods

All statistical analyses will be performed independently within the UMBD under the responsibility of Professor A Duhamel. Analyses will be performed using the SAS 9.4 or higher software (SAS Institute Inc). Characteristics of the patients at baseline will be described; qualitative variables will be described by numbers and percentages, and quantitative variables will be described either by the mean and standard deviation for Gaussian distribution or by the median and interquartile range (ie, 25th and 75th percentiles). The normality of the distributions will be tested by a Shapiro-Wilk test and checked graphically using histograms.

To answer to the main objective, the rejection rules of Simon’s optimal plan will be used. According to Simon’s optimal plan, after including 8 patients in a first step, the experimental treatment (PDT treatment with the PAGETEX device) will be rejected if the number of patients with disease control at 3 months (assessed by the investigator) is less than or equal to 3. If the trial continues in the second stage, the experimental treatment will be rejected if, out of the 24 patients, the number of patients with disease control at 3 months is less than or equal to 10. The 95% bilateral confidence interval of the 3-month disease control rate will be calculated. The control rates of the disease and its 95% confidence interval, as assessed by the investigating physician and the independent committee, will be calculated. Changes in the quality of life, sexual quality of life, anxiety, and depression measured by the DLQI, HADS, FSFI, and SF-36 questionnaires will be estimated using a mixed linear model (covariance pattern ) including time (inclusion visit, visit at 3 months, visit at 6 months) as the fixed effect. The pain experienced measured by the visual analog scale will be described as a continuous variable and as a qualitative variable to 4 classes. Severity of the erythema assessed by a 4-point ordinal scale will be described at each time (inclusion, 3 months, and 6 months). Severity of the erythema evaluated at follow-up visits will be compared to severity of the erythema evaluated at the inclusion visit using the Wilcoxon signed-rank test. Percentages of patients with a decrease in severity grade at 3 months and 6 months compared with baseline will be described. Evolution of the severity of the erythema quantified by a colorimetry will be evaluated by the same method. The presence of fluorescence in Paget cells after each PDT session will be described to prove the production of PpIX. The link between the presence of fluorescence after each PDT session and the disease control rates at 3 months and 6 months will be studied using a Fisher exact test. Finally, rates of patients with at least one Paget cell in the thickness of the epidermis biopsies, patient satisfaction questionnaires scores, and frequencies of adverse events will be described.

### Ethical Considerations

The trial is conducted in accordance with principles enunciated in the Declaration of Helsinki, as well as International Council for Harmonisation guidelines and article L1121-4 of the French Health Code. The study protocol has been submitted for review and approval by the French Ethics Committee (2018-68) and the French National Agency for the Safety of Medicines and Health Products (2018-A01873-52 and 2018-002604-13). The trial was registered at ClinicalTrials.gov [NCT03713203]. The investigator must ensure that subjects are informed clearly and fully about the purpose, potential risks, and other critical issues regarding clinical studies in which they volunteer to participate. Freely given written informed consent must be obtained from each subject prior to clinical study participation, including informed consent for any screening procedures conducted to establish subject eligibility for the study. The rights, safety, and well-being of the subjects are the most important considerations and should prevail over interests of science and society.

### Patient Confidentiality and Involvement

Patients or the public were not involved in the conceptualization or carrying out of this research. Concerning treatment of personal data, this study is in compliance with the French methodology of reference (MR0001), which is a simplified declaration of data from medical research to the French National Data Protection Authority. The only persons authorized to access data and modify files generated by the study will be persons directly involved in the study. The participants will have access to the data and be able to modify them at any moment through one of the referring investigators of the study. The sponsor affirms the patient’s right to protection against invasion of privacy.

## Results

The first patient was included in September 2019. Evaluation of disease control in the first 8 patients will determine the study's continuation (early 2020). The final visit of the last patient is expected to August 2022. Analysis of the results is scheduled for the end of 2022, and results are expected to be published at the beginning of 2023.

## Discussion

PDT using 5-aminolevulinic acid has been employed sporadically to treat EMPV even if it is not the common treatment of this pathology [15,16]. MAL-PDT is a relatively simple procedure to treat large and multiple lesions and can be repeated without functional or cosmetics effects. This clinical trial is the only trial registered on ClinicalTrials.gov that investigates the efficacy and safety of PDT in EMPV. But its boundaries are that disease evolution is difficult to evaluate because symptom improvement is based on a medical opinion by comparison of photographs and the limitation of the penetration of MAL at a depth of around 2 to 3 mm for a wavelength of 630 nm according to the thickness of the lesions. In case of positive results, a randomized trial comparing PDT with imiquimod will be performed.

## Abbreviations

5-ALA: 5-aminolevulinic acid
DLQI: Dermatology Life Quality Index
EMPV: extramammary Paget disease of the vulva
FSFI: Female Sexual Functioning Index
HADS: Hospital Anxiety and Depression Scale
LEF: light-emitting fabric
MAL: methyl aminolevulinate
PDT: photodynamic therapy
PpIX: protoporphyrin IX
PS: photosensitizing molecule
SF-36: 36-item Short Form Health Survey
UMBD: Unit of Methodology, Biostatistics and Data Management
